# HISPANIC DEMENTIA CAREGIVERS INVOLVEMENT IN EVERYDAY DECISION-MAKING

**DOI:** 10.1093/geroni/igae098.3286

**Published:** 2024-12-31

**Authors:** Abigail Poe, Loreli Alvarez, Danny Wang, Natashia Bibriescas, Frank Puga

**Affiliations:** University of Alabama at Birmingham, Birmingham, Alabama, United States; University of Alabama at Birmingham, Birmingham, Alabama, United States; The Pennsylvania State University, University Park, Pennsylvania, United States; The University of Texas Austin, Austin, Texas, United States; University of Alabama at Birmingham, Birmingham, Alabama, United States

## Abstract

This analysis aimed to examine the degree of decision-making involvement (DMI) of Hispanic and Latinx (H&L) dementia caregivers of people living with dementia (PLWD). Data was used from an ongoing study on the daily mental health experiences of H&L caregivers of PLWD, entitled “Nuestros Días” (Our Days). Analysis of variance was used to examine differences in caregiver involvement in everyday decisions, such as determining meals (Decision-Making Involvement Scale), based on H&L dementia caregiver age (e.g., 40-49, 50-59, etc.). A subset of 47 H&L dementia caregivers from the main study who completed the DMI scale were included in the analysis. The majority of the H&L caregivers identified as female (91.3%), with a mean age of 47 (SD = 17.4). Around half (48.6%) of the H&L caregivers were working full-time (30+ hours a week). H&L dementia caregivers reported moderate involvement (M = 22.9, SD = 7.1, Range 0-36) in DMI for the PLWD. Caregiver age was significantly associated with DMI (F(3, 40) = 3.08, p =.03). Post hoc comparisons using the Tukey HSD test indicated that the mean DMI for caregivers in the 60-69 age group was significantly higher than the mean DMI for caregivers in the 70+ age group (MD = 11.05, SD = 2.93, p =.027). Age may influence H&L caregiver DMI for the PLWD. Further studies are needed to identify additional factors (e.g., contextual, cultural, contextual) associated with H&L dementia caregiver’s DMI for the PLWD and potential associations with caregiver stress and burden.

